# Assessment of Duplicate Evidence in Systematic Reviews of Imaging Findings of Children With COVID-19

**DOI:** 10.1001/jamanetworkopen.2020.32690

**Published:** 2021-01-07

**Authors:** Giordano Pérez-Gaxiola, Francisca Verdugo-Paiva, Gabriel Rada, Iván D. Flórez

**Affiliations:** 1 Sinaloa Pediatric Hospital’s Cochrane Associate Centre, Culiacan, Mexico; 2 Epistemonikos Foundation, Santiago, Chile; 3 Department of Pediatrics, University of Antioquia, Medellin, Colombia

## Abstract

This cross-sectional study maps a coronavirus research question to illustrate the overlap and shortcomings of the evidence syntheses in this area.

## Introduction

Formulating evidence-based recommendations for children affected by severe acute respiratory syndrome coronavirus 2 (SARS-CoV-2) is challenging. Identifying and synthesizing the evidence to inform these recommendations has become difficult. With the explosion of publications on preprint servers and in journals, waste in coronavirus disease 2019 (COVID-19) research is common. While replication of systematic reviews may be appropriate in some instances, duplication refers to needless repetition of the same review.^1^ Answering simple questions, such as the most common findings in children with COVID-19, requires an enormous effort. We aimed to map 1 of these questions (ie, what is the spectrum and frequency of imaging findings in children with COVID-19?) to illustrate the overlap and shortcomings of the evidence syntheses in this area.

## Methods

This cross-sectional study began with systematic searches in the Living Overview of Evidence platform for COVID-19, a system that maps population, intervention, comparison, and outcome (PICO) questions to a repository maintained through regular searches in more than 40 sources, including databases, preprint servers, trial registries, and others. An artificial intelligence algorithm maintains the repository, and the information is transmitted in real time. Search methods for COVID-19 in the Living Overview of Evidence are detailed in the eAppendix in the Supplement. This study followed the Preferred Reporting Items for Systematic Reviews and Meta-analyses (PRISMA) reporting guideline, modified for overviews. Because this was a secondary analysis of already-published articles, no institutional review board approval was sought.

We included systematic reviews describing imaging findings in children younger than 18 years (excluding neonates) with SARS-CoV-2 infection and primary studies with more than 30 children included. Two reviewers (G.P.-G. and F.V.-P.) independently evaluated potentially eligible studies. Searches had no language restrictions and covered the period until September 1, 2020.

We built a matrix of evidence using Epistemonikos Database (Epistemonikos) to compare the studies included in the reviews. A matrix of evidence is a table displaying all systematic reviews answering the same question as well as the studies answering the question of interest included in these reviews. We provide a narrative description of the results. No statistical testing was conducted.

## Results

We identified 25 systematic reviews, including 17 primary studies, answering the question of interest (Figure). Only 6 of the 25 systematic reviews identified (24%) had been previously registered in PROSPERO or other registries. The number of primary studies identified by each particular review ranged from 1 to 9. Our search found 11 eligible primary studies that were not identified by any of the reviews.

**Figure.**
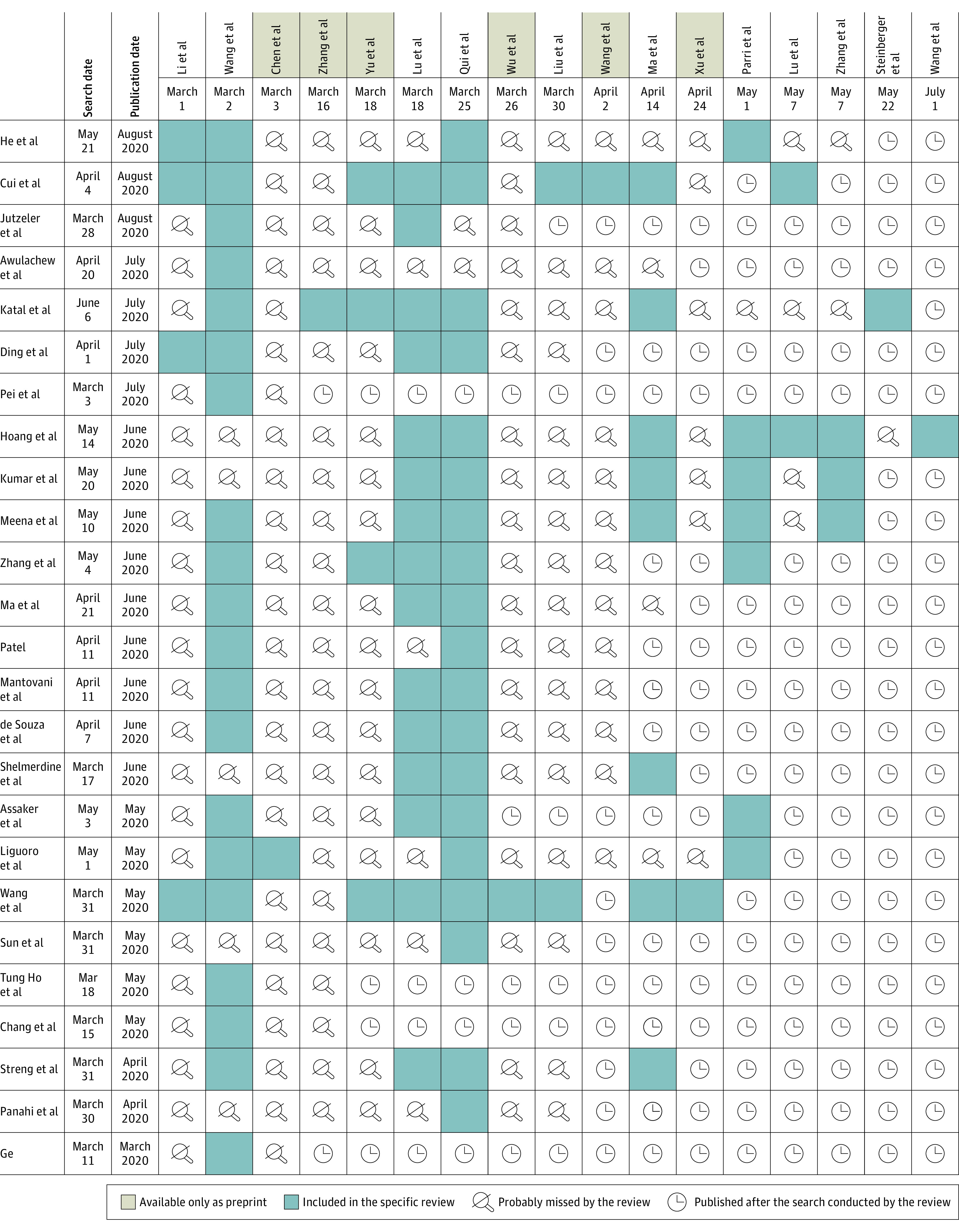
Matrix of Evidence Displaying the Number of Systematic Reviews and Primary Studies Included in Each Review References can be found in the eReferences in the Supplement.

The most recent review with the largest number of included studies (Cui et al^2^) had only 9 of 28 articles (32%) that were eligible according to our analysis. We explored whether this review explicitly excluded these studies or whether there were additional criteria explaining why they were not included. Four studies (21%) were probably missed during the review process, and the other 15 (79%) were published after their search.

## Discussion

This study presents a particular case in which, in less than 6 months, the literature was flooded with more systematic reviews than primary studies trying to answer a very specific clinical question, such as the imaging findings in children with COVID-19. Replication of systematic reviews may be appropriate to verify their findings or to extend or narrow the question they are trying to answer.^1^ However, needless repetition is wasteful. Initiatives like the PROSPERO database were created so authors could identify ongoing systematic reviews and perhaps stop the development of a new, unnecessary study.^3,4^

Duplication at a massive level, which has happened with COVID-19, is unjustified and may be unethical.^1,5,6^ None of the systematic reviews included the totality of primary studies, which may be partly explained by the rapid rate of reporting of new studies but also by limitations of search strategies. This also highlights how quickly published reviews can become obsolete if they are not continuously updated.

This study has limitations. Our analysis cannot provide clinical guidance regarding the imaging findings of children with COVID-19. The findings are also limited to the date of our search.

## Notes

Supplement. **eAppendix.** Supplementary Methods **eReferences.**
