# Supplementary material for: Assessment of Duplicate Evidence in Systematic Reviews of Imaging Findings of Children With COVID-19
Source: JAMA Netw Open. 2021 Jan 7;4(1):e2032690. doi: 10.1001/jamanetworkopen.2020.32690 (PMC7791356; doi:10.1001/jamanetworkopen.2020.32690)
Supplement: Supplement. — eAppendix. Supplementary Methods eReferences. [file jamanetwopen-e2032690-s001.pdf]

Supplemental Online Content

Pérez-Gaxiola G, Verdugo-Paiva F, Rada G, Flórez ID. Assessment of duplicate evidence in systematic reviews of imaging findings of children with COVID-19. *JAMA Netw Open*. 2021;4(1):e2032690. doi:10.1001/jamanetworkopen.2020.32690

eAppendix. Supplementary Methods
eReferences

This supplemental material has been provided by the authors to give readers additional information about their work.

© 2021 Pérez-Gaxiola et al. *JAMA Network Open.*

eAppendix. Supplementary Methods

Methods of the Special L·OVE of Coronavirus (COVID-19)

The Living OVerview of Evidence (L·OVE) builds upon the general methods of the L·OVE platform and incorporates the following new methods:

Search methods

Search strategy

All of the evidence organized in the L·OVE platform is retrieved in real-time from the Epistemonikos Database (See Epistemonikos Database methods here).

The team maintaining the L·OVE platform devised a search strategy for COVID-19, and each of the individual PICO questions available, using the following approach:

- Identification of terms relevant to the population and intervention/test/variable components of the search strategy, applying Word2vec technology to the corpus of documents available in Epistemonikos Database.
- Discussion of terms with content and methods experts to identify relevant, irrelevant and missing terms.
- Creation of a sensitive boolean strategy encompassing all the relevant terms
- Iterative analysis of articles missed by the boolean strategy, and refinement of the strategy accordingly.

The following strategy was used to search for Coronavirus infection (COVID-19 and other coronavirus infections affecting humans):

© 2021 Pérez-Gaxiola et al. *JAMA Network Open.*

coronavir* OR coronovirus* OR betacoronavir* OR "beta-coronavirus" OR "beta-coronaviruses" OR "corona virus" OR "virus corona" OR "corono virus" OR "virus corono" OR hcov* OR covid* OR "2019-ncov" OR cv19* OR "cv-19" OR "cv 19" OR "n-cov" OR ncov* OR (wuhan* and (virus OR viruses OR viral)) OR sars* OR sari OR "severe acute respiratory syndrome" OR mers* OR "middle east respiratory syndrome" OR "middle-east respiratory syndrome" OR "2019-ncov-related" OR "cv-19-related" OR "n-cov-related"

Search sources

To complement the searches in the 10 sources routinely performed in Epistemonikos Database (See Epistemonikos Database methods here), we conduct searches in the following sources:

Pubmed/medline (updated several times a day)

EMBASE (updated weekly)

CINAHL (updated weekly)

PsycINFO (updated weekly)

LILACS (Latin American & Caribbean Health Sciences Literature) (updated weekly)

Wanfang Database (updated every 2 weeks)

CBM - Chinese Biomedical Literature Database (updated every 2 weeks)

CNKI - Chinese National Knowledge Infrastructure (updated every 2 weeks)

VIP - Chinese Scientific Journal Database (updated every 2 weeks)

IRIS (WHO Institutional Repository for Information Sharing) (updated weekly)

IRIS PAHO (PAHO Institutional Repository for Information Sharing)) (updated weekly)

IBECS - Índice Bibliográfico Español en Ciencias de la Salud (Spanish Bibliographic Index on Health Sciences) (updated weekly)

Microsoft Academic (last searched: Sept 4, 2020)

ICTRP Search Portal (updated daily)

© 2021 Pérez-Gaxiola et al. *JAMA Network Open.*

Clinicaltrials.gov (updated daily)

ISRCTN registry (updated daily)

Chinese Clinical Trial Registry (updated daily)

IRCT - Iranian Registry of Clinical Trials (updated daily)

EU Clinical Trials Register: Clinical trials for covid-19 (updated daily)

NIPH Clinical Trials Search (Japan) - Japan Primary Registries Network (JPRN) (JapicCTI, JMACCT CTR, jRCT, UMIN CTR) (updated daily, via ICTRP search portal)

UMIN-CTR - UMIN Clinical Trials Registry (updated daily, via ICTRP search portal)

JRCT - Japan Registry of Clinical Trials (updated daily, via ICTRP search portal)

JAPIC Clinical Trials Information (updated daily, via ICTRP search portal)

Clinical Research Information Service (CRiS), Republic of Korea (updated daily, via ICTRP search portal)

ANZCTR - Australian New Zealand Clinical Trials Registry (updated daily, via ICTRP search portal)

ReBec - Brazilian Clinical Trials Registry (updated daily, via ICTRP search portal)

CTRI - Clinical Trials Registry - India (updated daily, via ICTRP search portal)

RPCEC - Cuban Public Registry of Clinical Trials (updated daily, via ICTRP search portal)

DRKS - German Clinical Trials Register (updated daily, via ICTRP search portal)

LBCTR - Lebanese Clinical Trials Registry (updated daily, via ICTRP search portal)

TCTR - Thai Clinical Trials Registry (updated daily, via ICTRP search portal)

NTR - The Netherlands National Trial Register (updated daily, via ICTRP search portal)

PACTR - Pan African Clinical Trial Registry (updated daily, via ICTRP search portal)

REPEC - Peruvian Clinical Trial Registry (updated daily, via ICTRP search portal)

SLCTR - Sri Lanka Clinical Trials Registry (updated daily, via ICTRP search portal)

medRxiv (updated several times a day)

© 2021 Pérez-Gaxiola et al. *JAMA Network Open.*

bioRxiv (updated several times a day)

SSRN Preprints (updated several times a day)

ChinaXiv (updated every 2 weeks)

SciELO Preprints (updated weekly)

Research Square (updated daily)

© 2021 Pérez-Gaxiola et al. *JAMA Network Open.*

eReferences

References of systematic reviews identified

S1. Chang TH, Wu JL, Chang LY. Clinical characteristics and diagnostic challenges of pediatric COVID-19: A systematic review and meta-analysis. Journal of the Formosan Medical Association = Taiwan yi zhi. 2020;119(5):982-989.

S2. Shelmerdine SC, Lovrenski J, Caro-Domínguez P, Toso S, Collaborators of the European Society of Paediatric Radiology Cardiothoracic Imaging Taskforce. Coronavirus disease 2019 (COVID-19) in children: a systematic review of imaging findings. Pediatric radiology. 2020;50(9):1217-1230.

S3. Patel NA. Pediatric COVID-19: Systematic review of the literature. American journal of otolaryngology. 2020;41(5):102573.

S4. Ding Y, Yan H, Guo W. Clinical Characteristics of Children With COVID-19: A Meta-Analysis. Frontiers in pediatrics. 2020;8:431.

S5. Jutzeler CR, Bourguignon L, Weis CV, Tong B, Wong C, Rieck B, Pargger H, Tschudin-Sutter S, Egli A, Borgwardt K, Walter M. Comorbidities, clinical signs and symptoms, laboratory findings, imaging features, treatment strategies, and outcomes in adult and pediatric patients with COVID-19: A systematic review and meta-analysis. Travel medicine and infectious disease. 2020;37:101825.
Jutzeler, Lucie Bourguignon, Caroline V. Weis,et al. Comorbidities, clinical signs and symptoms, laboratory findings, imaging features, treatment strategies, and outcomes in adult and pediatric patients with COVID-19: A systematic review and meta-analysis. medRxiv. 2020;

S6. Awulachew E, Diriba K, Anja A, Getu E, Belayneh F. Computed Tomography (CT) Imaging Features of Patients with COVID-19: Systematic Review and Meta-Analysis. Radiology research and practice. 2020;2020:1023506.

S7. Tung Ho CL, Oligbu P, Ojubolamo O, Pervaiz M, Oligbu G. Clinical Characteristics of Children with COVID-19. AIMS public health. 2020;7(2):258-273.

S8. Ge Yanling. Systematic review of novel coronavirus pneumonia epidemiology, clinical features and discharge outcomes in children based on 8 case series and 10 case reports. 中国循证儿科杂志 (Chinese Journal of Evidence Based Pediatrics). 2020;1(15).

S9. Pei Y, Liu W, Masokano IB, Li F, Xie S, Zhou G, Long X, Liao W, Hu S, Li W. Comparing Chinese children and adults with RT-PCR positive COVID-19: A systematic review. Journal of infection and public health. 2020;13(10):1424-1431.

S10. Streng A, Hartmann K, Armann J, Berner R, Liese JG. [COVID-19 in hospitalized children and adolescents]. Monatsschrift Kinderheilkunde : Organ der Deutschen Gesellschaft fur Kinderheilkunde. 2020;:1-12

S11. Sun Z, Zhang N, Li Y, Xu X. A systematic review of chest imaging findings in COVID-19. Quantitative imaging in medicine and surgery. 2020;10(5):1058-1079.

S12. Wang Z. et al

Wang Z., Ma Y., Xun Y. et al. Clinical characteristics of children with COVID-19: A rapid review and meta-analysis. Annals of Translational Medicine. 2020;8(10):620.

Wang Z, Qi Zhou, Chenglin Wang et al. Clinical Characteristics of Children with COVID-19: A Rapid Review and Meta-Analysis. medRxiv. 2020;

S13. de Souza TH, Nadal JA, Nogueira RJN, Pereira RM, Brandão MB. Clinical Manifestations of Children with COVID-19: a Systematic Review. Pediatric pulmonology. 2020;55(8):1892-1899.

de Souza, Nadal JA, Nogueira RJN et al. Clinical Manifestations of Children with COVID-19: a Systematic Review. medRxiv. 2020;

© 2021 Pérez-Gaxiola et al. *JAMA Network Open.*

S14. Cui X, Zhao Z, Zhang T, Guo W, Guo W, Zheng J, Zhang J, Dong C, Na R, Zheng L, Li W, Liu Z, Ma J, Wang J, He S, Xu Y, Si P, Shen Y, Cai C. A systematic review and meta-analysis of children with Coronavirus Disease 2019 (COVID-19). Journal of medical virology. 2020;

S15. Zhang L, Peres TG, Silva MVF, Camargos P. What we know so far about Coronavirus Disease 2019 in children: A meta-analysis of 551 laboratory-confirmed cases. Pediatric pulmonology. 2020;55(8):2115-2127.

S16. Mantovani A, Rinaldi E, Zusi C, Beatrice G, Saccomani MD, Dalbeni A. Coronavirus disease 2019 (COVID-19) in children and/or adolescents: a meta-analysis. Pediatric research. 2020;

S17. Liguoro I, Pilotto C, Bonanni M et al. SARS-COV-2 infection in children and newborns: a systematic review. European journal of pediatrics. 2020;179(7):1029-1046.

S18. Assaker R, Colas AE, Julien-Marsollier F et al. Presenting symptoms of COVID-19 in children: a meta-analysis of published studies. British journal of anaesthesia. 2020;125(3):e330-e332.

S19. Katal S, Johnston SK, Johnston JH, Gholamrezanezhad A. Imaging findings of SARS-CoV-2 infection in pediatrics: A systematic review of coronavirus disease 2019 (COVID-19) in 850 patients. Academic radiology. 2020;27(11):1608-1621.

S20. Kumar J, Meena J, Yadav A, Yadav J. Radiological Findings of COVID-19 in Children: A Systematic Review and Meta-Analysis. Journal of tropical pediatrics. 2020;

S21. Panahi L, Amiri M, Pouy S. Clinical Characteristics of COVID-19 Infection in Newborns and Pediatrics: A Systematic Review. Archives of academic emergency medicine. 2020;8(1):e50.

S22. Ma X, Liu S, Chen L, Zhuang L, Zhang J, Xin Y. The clinical characteristics of pediatric inpatients with SARS-CoV-2 infection: a meta-analysis and systematic review. Journal of medical virology. 2020;

© 2021 Pérez-Gaxiola et al. *JAMA Network Open.*

S23. He Y, Tang J, Zhang M, Wang HR, Li WX, Xiong T, Li YP, Mu DZ. [Clinical features of coronavirus disease 2019 in children: a systemic review of severe acute respiratory syndrome, Middle East respiratory syndrome, and coronavirus disease 2019]. Zhongguo dang dai er ke za zhi = Chinese journal of contemporary pediatrics. 2020;22(8):844-853.

S24. Meena J, Yadav J, Saini L, Yadav A, Kumar J. Clinical Features and Outcome of SARS-CoV-2 Infection in Children: A Systematic Review and Meta-analysis. Indian pediatrics. 2020;

S25. Hoang A., Chorath K., Evans M., Burmeister-Morton F., Burmeister F., Naqvi R., Petershack M., Moreira A., Moreira A.. COVID-19 in 7780 pediatric patients: A systematic review. EClinicalMedicine. 2020;24:100433.

References of primary studies included in systematics reviews

S19. Xu H, Liu E, Xie J et al. A follow-up study of children infected with SARS-CoV-2 from Western China. medRxiv. 2020;

S20. Steinberger S, Lin B, Bernheim A, Chung M, Gao Y, Xie Z, Zhao T, Xia J, Mei X, Little BP. CT Features of Coronavirus Disease (COVID-19) in 30 Pediatric Patients. AJR. American journal of roentgenology. 2020;:1-9.

S21. Ma H et al.

Ma H, Hu J, Tian J, Zhou X, Li H, Laws MT, Wesemann LD, Zhu B, Chen W, Ramos R, Xia J, Shao J. A single-center, retrospective study of COVID-19 features in children: a descriptive investigation. BMC medicine. 2020;18(1):123.

Ma H, Hu J, Tian J, et al. Visualizing the novel coronavirus (COVID-19) in children: what we learn from patients at Wuhan children’s hospital. SSRN Journal. 2020;

S22. Zhang C, Gu J, Chen Q, Deng NA et al. Clinical Characteristics of 34 Children with Coronavirus Disease-2019 in the West of China: a Multiple-center Case Series. medRxiv. 2020;

© 2021 Pérez-Gaxiola et al. *JAMA Network Open.*

S23. Liu J, Luo W, Deng Z et al.. Clinical and epidemiological characteristics of 91 children conformed with COVID-19. 中华医院感染学杂志 (Chinese Journal of Nosocomiology). 2020;30:1645-9.

S24. Yu H, Cai Q, Dai X et al. The clinical and epidemiological features and hints of 82 confirmed COVID-19 pediatric cases aged 0-16 in Wuhan, China. medRxiv. 2020;

S25. Wang D, Ju XL, Xie F, et al. [Clinical analysis of 31 cases of 2019 novel coronavirus infection in children from six provinces (autonomous region) of northern China]. 中华儿科杂志 (Chinese journal of pediatrics). 2020;58(4):E011.

S26. Qiu H, Wu J, Hong L et al. Clinical and epidemiological features of 36 children with coronavirus disease 2019 (COVID-19) in Zhejiang, China: an observational cohort study. The Lancet. Infectious diseases. 2020;20(6):689-696.

S27. Wu Q, Yuhan Xing, Lei Shi et al. Epidemiological and Clinical Characteristics of Children with Coronavirus Disease 2019. medRxiv. 2020;

S28. Zhang B, Liu S, Zhang J et al. Children hospitalized for coronavirus disease 2019 (COVID-19): a multicenter retrospective descriptive study. The Journal of infection. 2020;81(2):e74-e75.

S29. Parri N, Lenge M, Buonsenso D, Coronavirus Infection in Pediatric Emergency Departments (CONFIDENCE) Research Group. Children with Covid-19 in Pediatric Emergency Departments in Italy. The New England journal of medicine. 2020;383(2):187-190.

S30. Li Q, Peng X, Sun Z, et al.. Clinical and imaging characteristics of children with coronavirus disease 2019 (COVID-19). 放射学实践 (Radiologic Practice). 2020;35(3):277-80.

S31. Wang Y, Zhu F, Wu J et al. Epidemiological and Clinical Characteristics of 74 Children Infected with SARS-CoV-2 in Family Clusters in Wuhan, China. SSRN. 2020;

© 2021 Pérez-Gaxiola et al. *JAMA Network Open.*

S32. Lu Y, Li Y, Deng W, Liu M et al. Symptomatic Infection is Associated with Prolonged Duration of Viral Shedding in Mild Coronavirus Disease 2019: A Retrospective Study of 110 Children in Wuhan. The Pediatric infectious disease journal. 2020;39(7):e95-e99.

S33. Wang Y, Zhu F, Wang C et al. The Risk of Children Hospitalized With Severe COVID-19 in Wuhan. The Pediatric infectious disease journal. 2020;39(7):e91-e94.

S34. Chen C, Cao M, Peng L, Guo X, Yang F, Wu W, Chen L, Yang Y, Yingxia L, Wang F. Coronavirus Disease-19 Among Children Outside Wuhan, China. SSRN. 2020;

S35. Lu X, Sun D, Ma YL et al.

a. Lu X, Zhang L, Du H, Zhang J et al. SARS-CoV-2 Infection in Children. The New England journal of medicine. 2020;382(17):1663-1665.
b. Ma YL, Xia SY, Wang M, Zhang SM, DU WH, Chen Q. [Clinical features of children with SARS-CoV-2 infection: an analysis of 115 cases]. Zhongguo dang dai er ke za zhi = Chinese journal of contemporary pediatrics. 2020;22(4):290-293.

References of primary studies not included in any of the identified systematic reviews

S37. Caro-Dominguez P, Shelmerdine SC, Toso S et al. Thoracic imaging of coronavirus disease 2019 (COVID-19) in children: a series of 91 cases. Pediatric radiology. 2020;50(10):1354-1368.

S38. Gaborieau L, Delestrain C, Bensaid P et al. Epidemiology and Clinical Presentation of Children Hospitalized with SARS-CoV-2 Infection in Suburbs of Paris. Journal of clinical medicine. 2020;9(7):1-10.

S39. Korkmaz MF, Türe E, Dorum BA et al. The Epidemiological and Clinical Characteristics of 81 Children with COVID-19 in a Pandemic Hospital in Turkey: an Observational Cohort Study. Journal of Korean medical science. 2020;35(25):e236.

© 2021 Pérez-Gaxiola et al. *JAMA Network Open.*

S40. Li Y, Wang H, Wang F, et al. Comparison of Hospitalized Patients with pneumonia caused by COVID-19 and influenza A in children under 5 years. International journal of infectious diseases : IJID : official publication of the International Society for Infectious Diseases. 2020;98:80-83.

S41. Mahmoudi S., Pourakbari B., Mamishi S. et al. The coronavirus disease 2019 (COVID-19) in children: A study in an Iranian children's referral hospital. Infect. Drug Resist.. 2020;13(13):2649-2655.

S42. Palabiyik F, Kokurcan SO, Hatipoglu N, et al. Imaging of COVID-19 pneumonia in children. The British journal of radiology. 2020;93(1113):20200647.

S43. Soltani J, Sedighi I, Shalchi Z et al. Pediatric coronavirus disease 2019 (COVID-19): An insight from west of Iran. Northern clinics of Istanbul. 2020;7(3):284-291.

S44. Sun D, Zhu F, Wang C, Wu J, Liu J, Chen X, Liu Z, Wu Z, Lu X, Ma J, Peng H, Xiao H. Children Infected With SARS-CoV-2 From Family Clusters. Frontiers in pediatrics. 2020;8(8):386.

S45. Xu H., Zhao R., Long X. et al. A follow-up study of children infected with SARS-CoV-2 from western China. Ann. Transl. Med.. 2020;8(10):623.

S46. Zhang C, Gu J, Chen Q, et al. Clinical and epidemiological characteristics of pediatric SARS-CoV-2 infections in China: A multicenter case series. PLoS medicine. 2020;17(6):e1003130.

S47. Zhang L, Huang S. Clinical Features of 33 Cases in Children Infected With SARS-CoV-2 in Anhui Province, China-A Multi-Center Retrospective Cohort Study. Frontiers in public health. 2020;8(8):255.

© 2021 Pérez-Gaxiola et al. *JAMA Network Open.*
